# Prevalence and barriers to health care transition for adolescent patients with childhood-onset chronic diseases across Japan: A nation-wide cross-sectional survey

**DOI:** 10.3389/fped.2022.956227

**Published:** 2022-09-01

**Authors:** Ikuho Sakurai, Mitsue Maru, Takako Miyamae, Masataka Honda

**Affiliations:** ^1^Department of Nursing, Faculty of Health Sciences, Saitama Prefectural University, Koshigaya, Japan; ^2^School of Nursing, College of Nursing Art and Science, University of Hyogo, Akashi, Japan; ^3^Department of Pediatric Rheumatology, Institute of Rheumatology, Tokyo Women's Medical University, Tokyo, Japan; ^4^Pediatric Nephrology, Tokyo Metropolitan Children's Medical Center, Tokyo, Japan

**Keywords:** childhood-onset chronic diseases, health care transition, transition program, barriers to transition, cross-sectional study, Japan

## Abstract

Since the Japan Pediatric Society published its “Recommendations on Transitional Care for Patients with Childhood-Onset Chronic Diseases” in 2014, there has been an increased interest in the health care transition of adolescents with childhood-onset chronic diseases in Japan. However, the actual status of healthcare transition was not studied yet. The purpose of this study was to explore the prevalence of transitional support for adolescent patients with childhood-onset chronic disease and the factors hindering their transition. We conducted an anonymous questionnaire survey in August 2020, targeting physicians and nurses involved in health care transition at 494 pediatric facilities in Japan. Survey items included demographic data, health care systems related to transition to adult departments, health care transition programs based on Six Core Elements (establishing transition policy, tracking and monitoring transition progress, assessing patient readiness for transition, developing the transition plan with a medical summary, transferring the patient, completing the transfer/following up with the patient and family), barriers to transition (34-item, 4-point Likert scale), and expectations in supporting transition (multiple-choice responses), which consisted of five items (78 questions); all questions were structured. Descriptive statistics were used for analysis. Of the 225 responses collected (45.5% response rate), 88.0% were from pediatricians. More than 80% of respondents transferred patients of 20 years or older, but only about 15% had took a structured transition process of four or more based on the Six Core Elements. The top transition barriers were “intellectual disability/rare disease” and “dependence on pediatrics” as patient/family factors, and “lack of collaboration with adult healthcare (relationship, manpower/system, knowledge/understanding)” as medical/infrastructure factors. The study provides future considerations, including the promotion of structured health care transition programs, development of transitional support tailored to the characteristics of rare diseases and disorders, and establishment of a support system with adult departments.

## Introduction

The health care transition (HCT) of adolescents with childhood-onset chronic disease from pediatric to adult health care systems has recently received worldwide attention. However, Japan is lagging behind other countries, as the concept of HCT was introduced only 10 years ago (1). The number of patients registered in the Research Project for Treatment of Specific Pediatric Chronic Diseases in Japan is approximately 93,000 per year (2). It is estimated that 95.7% of patients with specific pediatric chronic diseases other than malignant neoplasms reach adulthood (3). However, these patients often develop complications in adulthood due to age-related changes in therapeutic areas, poor treatment adherence, and the development of lifestyle-related diseases (4–6). Therefore, there is a need to transition from the pediatric to the adult health care system; a smooth transition of these patients to the adult health care system appropriate to their needs, where they can receive appropriate medical care and the life they desire, is needed. Thus, the implementation of a structured transition program that includes support for patient independence is recommended (7, 8). In Japan, in 2014, the Japan Pediatric Society published a consensus statement on the “Proposal for Transitional Care for Patients with Childhood-Onset Diseases” (9).

Clinical Reporting in Transitional Care, recommended by the American Academy of Pediatrics, the American Academy of Family Physicians, and the American Board of Internal Medicine (7), uses Got Transition® (10). The Six Core Elements of the National Resource Center on Health Care Transitions specifically describe both pediatric and adult health care programs and include the following: (1) establishing the transition policy, (2) tracking and monitoring transition progress, (3) assessing the patient's readiness for transition, (4) developing the transition plan with a medical summary, (5) transferring the patient, (6) completing the transfer and following up with the patient and family. These consist of and define the basic elements in a structured transition process.

Some of the questions in this study were developed based on these six elements. Other countries have reported outcomes of transition support using the Six Core Elements (11, 12). In addition, several literature reviews on health care transitions (13–16) also report measuring and evaluating the outcomes of transition interventions and transition models, describing the effectiveness of implementing structured transition programs. In Japan, transition support using the Six Core Elements was implemented in five hospitals under the Ministry of Health, Labor and Welfare's Transition Support Model Project (17, 18). Later, in FY2019, the Ministry of Health, Labor and Welfare, Research Group created the Six Core Guides for Adult Transition Support (19) based on the Six Core Elements and distributed it to children's hospitals nationwide. However, it is unclear to what extent transition support in Japan is consistent with the programs recommended in the United States and Japan.

In Japan, 62.3% of patients with childhood-onset chronic diseases, aged 20 and older, regularly visited multiple medical facilities, with pediatrics being the primary department of care for about half of them (20), indicating that the transition to adult care is not smooth (21). Patients with childhood-onset chronic diseases are required to transfer from the medical cost subsidy system under the measures for specific pediatric chronic diseases to the designated intractable diseases system if they meet the criteria for disease severity (22). However, patients who do not meet some of the criteria are currently forced to bear the long-term burden of high medical costs or to forgo the best treatment because eligibility for the public medical cost subsidy system ceases after the age of 20 (21).

The age of 20, when the subsidy for medical expenses for pediatric chronic diseases is no longer available, is a major turning point in the lives of patients as they are at a crossroads in terms of employment and higher education. However, studies of Japanese patients with pediatric chronic diseases have reported that patients with chronic pediatric diseases face difficulties in earning a living on their own due to low income and low employment (20). Thus, increased health care costs can be burdensome for those with low levels of education and limited employment opportunities due to pediatric chronic illnesses. Particularly, patients who are unable to cope with these increased health care costs will have no choice but to give up appropriate medical care.

Reported barriers to transition include lack of knowledge of pediatric-specific conditions and understanding of adolescent patients and families by adult departments, fear of losing trust and longstanding relationships with pediatricians, difficulty finding adult providers, fundamental differences between pediatric and adult care, and negative beliefs and expectations of adult departments (12, 23, 24). In Japan, the high dependence of patients and families on pediatrics, lack of awareness among health care professionals, and anxiety and distrust of adult medicine have been noted (25). In addition, although there have been recent health care provider surveys of general pediatric nurses and adult nurses (26), there is no updated national survey on transition barriers among health care providers who are central to transitional care in Japan. Therefore, we believe that there is an urgent need to understand the actual status of transitional care and investigate barriers to transition nationwide so that all patients with childhood-onset chronic diseases can smoothly transition to adult health care systems and receive appropriate medical care.

We believe that the results of this study will contribute to clarifying the role of such programs, improving the quality of transition support at medical institutions, and establishing a system that can support the transition to independence for patients. To this end, this study aimed to explore the status (prevalence) of transition support for adolescent patients in pediatric institutions and the factors, issues, and challenges in the health care system that hinder HCT.

## Materials and methods

### Definition of terms

Pediatric chronic diseases: chronic diseases that occur in children under 15 years.

Transition support: assistance for transition from the pediatric health care system to the adult health care system.

### Research design

This was a cross-sectional survey study of physicians and nurses providing transition support at pediatric institutions in Japan.

### Setting

The study was conducted between August to November 2020. We sent pen-and-paper questionnaires to 494 medical institutions in Japan that specialize in pediatrics (pediatric specialty hospitals, pediatric cancer center hospitals, hospitals with specific functions that provide pediatric care, university hospitals, general hospitals, hospitals that provide home medical care for children, comprehensive perinatal care centers, regional perinatal care centers, etc.).

### Participants

The eligibility criteria were physicians or nurses working at the above facilities and providing transition assistance at the time of the survey. The facility director selected respondents who met the eligibility criteria and gave them a survey form. Participants were assumed to have completed and directly returned the unmarked questionnaire themselves.

### Variables, data sources, and measurement methods

These items were generated based on a literature review, expert discussion, and interviews with three CNS in pediatrics (27). A Pilot Study was completed by sending the questionnaire to two pediatricians of HCT experts for content validity testing. Face validity was also tested on three similar participants. Alterations were made regarding feedback from the Pilot Study, such as changing the questions that were considered ambiguous. Then we formed the final version of the questionnaire for distribution to participants.

This study was in the initial discovery phase regarding actual transition support. The following data were included:

(1) Demographic data: information about the participant (job title, specialty, position, years of experience supporting transition, location of transition support) and information outlining the institution (type of founding agencies, disease groups treated).
(2) Medical care system for transfer to adult departments: medical care system for adolescent patients with chronic diseases (implementation of adolescent patient transfers, availability of specialized outpatient clinics and dedicated personnel, age at which the HCT program starts, reasons for starting support, collaboration with community family physicians, collaboration with adult hospitals and departments, use of educational and support tools for transition support).
(3) Contents of the HCT program based on the Six Core Elements (10): establishing the transition policy; tracking and monitoring transition progress; assessing the patient's readiness for transition; developing the transition plan with a medical summary; transferring the patient; completing the transfer and following up with the patient and family.
(4) Barriers to transition from pediatric to adult care (13, 18, 19, 23–32): based on 11 items for patients, nine for family members, and 14 for health care providers, the survey respondents' self-reported factors hindering the transition to adult care. A 4-point Likert scale was used for each item with the following response options: not at all applicable, not very applicable, fairly applicable, and very applicable.
(5) Transitional support for adolescent patients that should be enhanced in the future (14–16, 18, 19, 25, 28–31): the survey provided the possibility of multiple-choice responses for each of the following items: policy and local government (four items); academic institutions (four items); affiliated facilities (eight items); and individuals (eight items).

### Bias

The questionnaire was anonymous to avoid bias in the participants' responses. The participants directly returned the questionnaires themselves.

### Quantitative variables

An average score of 1 (not applicable at all), 2 (not very applicable), 3 (fairly applicable), and 4 (very applicable) was extracted to measure the factors preventing transition to adult care.

### Data analysis methods

Descriptive statistics including frequencies, means, and standard deviations were used in the analysis.

### Ethical considerations

When selecting potential research collaborators, return envelopes were distributed only to research participants to avoid the exercise of coercive power, the disclosure to outside parties of the presence or absence of replies, or the contents of the questionnaire. The questionnaires were unsigned to prevent the identification of the participants, and their responses to the questionnaire indicated that they had agreed to cooperate in the research. This study was conducted after obtaining approval from the ethical review committee of the researcher's institution (No. 19019).

## Results

A total of 494 copies of the questionnaire were distributed, and 225 were collected (45.5% response rate). To address missing data, statistical analyses were conducted using only valid responses in each section.

### Overview of the respondents

The respondents included 199 (88.4%) physicians and 26 (11.6%) nurses, with 22 (9.8%) children's hospital respondents among them. A total of 153 (68.0%) had more than 10 years of clinical experience in transition support, 179 (79.5%) were in administrative positions, and 76 (33.8%) were Specialized Physicians or Certified Nurses/Clinical Nurse Specialists. The following were multiple responses: the places of care delivery for adolescents with chronic diseases included outpatient pediatrics clinics 204 (90.7%); neuromuscular diseases were the most common with 116 cases (51.6%), followed by syndromes involving chromosomal or genetic changes with 104 cases (46.2%) (Table 1).

**Table 1:** Participants background (*n* = 225).

	**No answer**	***n***	**%**
Sex	2		
Male		169	75.1
Profession	0		
Pediatrician		199	88.4
Nurse		26	11.6
Hospital	0		
Children's Hospital		22	9.8
Pediatrics other than Children's Hospital		203	90.2
Administrative Position	0		
Yes		179	79.5
Certified or specialization	0		
Specialized physicians		64	28.4
Certified nurses/clinical nurse specialists		12	5.3
No		149	66.2
Total years of experience supporting transition	5		
1 <		6	2.7
1–3		20	8.9
4–9		41	18.2
≧10		153	68.0
Place of care delivery for teens with chronic disease (multiple responses)			
Specialization clinic		5	2.2
Center		3	1.3
Outpatient clinic pediatrics		204	90.7
Inpatient pediatrics		51	22.7
Other		9	4.0
Specialization (multiple responses)			
Neuro-muscle		116	51.6
Syndromes involving chromosomal or genetic changes		104	46.2
Endocrine		81	36.0
Childhood cancer		71	31.6
Cardiology		68	30.2
Respiratory		60	26.7
Type 1 DM		58	25.8
Kidney		56	24.9
Congenital/Inherited metabolic diseases		51	22.7
Other		185	82.2

### Medical care system for transfer to adult departments

Twenty (10.0%) had a specialty outpatient clinic and 61 (30.5%) had a full-time person in charge. The age at which the HCT program started was stated by 36 respondents (16.0%). Academic/career change and age were the most common reasons for starting support services, with 155 respondents (68.9%), followed by diseases outside the scope of pediatricians, with 135 (60.0%). Regarding collaboration with other agencies and departments, 80 (40.0%) respondents indicated that they collaborated with the local family physician, while 97 (48.5%) indicated that they collaborated with adult hospitals and departments. A total of 159 (79.5%) reported no use of educational and support tools for transition assistance, indicating that the guides were not widely used (Table 2).

**Table 2:** Medical care system for transfer to adult departments (*n* = 200).

	**No answer**	***n***	**%**
Specialized clinic	1		
Yes		20	10.0
Specialist	33		
Yes		61	30.5
Define the age for starting the HCT program			
Yes		36	16.0
Not defined		163	72.4
Other		1	0.4
Reason to start the HCT program (multiple responses)			
Academic/carrier change		155	68.9
Age		155	68.9
Disease outside the scope of pediatricians		135	60.0
Patients' preference		93	41.3
Family's preference		73	32.4
Psycho-social maturity		71	31.6
Pediatrician's circumstances		67	33.5
Stable disease condition		62	27.6
Collaboration with general practitioner in community	1		
Yes		80	40.0
Adult practitioner		69	34.5
Child practitioner		24	12.0
No		119	59.5
Collaboration with the adult practitioner in the hospital	0		
Yes		97	48.5
Educational/information package for transition	1		
Yes original		13	6.5
Yes use the existing package		25	12.5
No		159	79.5
Other		2	1.0

HCT, health care transition.

### Contents of the HCT program based on the six core elements

Two hundred respondents indicated that they transferred adolescent patients to adult departments, with “transferring the patient” being the most common response. Of the transition planning, 155 (77.5%) of the respondents prepared medical summaries. Medical summaries included: disease name with 81 cases (40.5%), examination results with 78 cases (39.0%), treatment summary with 77 cases (38.5%), and prescribed medicine/care with 74 cases (37.0%).

More than 70% responded “No” to all five other content areas: establishing the transition policy with 180 (90%), tracking and monitoring transition progress with 179 (89.5%), assessing the patient's readiness for transition with 143 (71.5%), developing the transition plan with 169 (84.5%), following up with the patient and family with 140 (70%), and patient feedback with 177 (89.4%).

For the combination of the Six Core Elements, 33 (16.5%) practiced only “transferring the patient,” 70 (35.0%) practiced “transferring the patient” and “making a medical summary,” and 30 (15.0%) practiced four or more elements (Table 3).

**Table 3:** Contents of the HCT program based on six core elements (*n* = 200).

	**No answer**	***n***	**%**
Combination of healthcare transition process based on the six core elements	0		
One element (Only “transferring the patient”)		33	16.5
Two elements		85	42.5
“Transferring the patient” and “making a medical summary”		70	35.0
Three elements		53	26.5
Four elements		13	6.5
Five elements		13	6.5
Six elements		4	2.0
Establishing the transition policy	0		
No		180	90
Yes		20	10.0
Tracking and monitoring transition progress	2		
No		179	89.5
Yes		16	8.0
Other		3	1.5
Assessing the patient's readiness for transition	0		
No		143	71.5
Yes		56	28.0
Use of assessment tools	1		
Yes		10	5.0
Evaluation Item (multiple responses)			
Understanding the disease		49	24.5
Need for the continuation of treatment		38	19.0
Medication adherence		38	19.0
Self-management		38	19.0
Employment and schooling		28	14.0
Treatment behavior		25	12.5
Cautionary points in daily life		24	12.0
Medical care system		24	12.0
Sexual and reproductive health		10	5.0
Other		1	0.5
Developing the transition plan with a medical summary			
Developing the transition plan	0		
No		169	84.5
Yes		30	15.0
Other		1	0.5
Making medical summary for transfer	1		
No		43	21.5
Yes		155	77.5
Disease name		81	40.5
Examination results		78	39.0
Treatment summary		77	38.5
Prescribed medicine/care		74	37.0
Emergency contact information		32	16.0
Explanatory document about the disease		31	15.5
Patient's self-management evaluation		9	4.5
Use of my medical history		8	4.0
Transition summary		4	2.0
Other		1	0.5
Transferring the patient	0		
Yes		200	100
Completing the transfer and following up with the patient and family			
Following up with the patient and family	0		
No		140	70.0
Yes		59	29.5
Other		1	0.5
Patient Feedback	2		
No		177	89.4
Yes		20	10.0
Other		1	0.5

### Barriers to transition from pediatric to adult care

The patient factors with the most scores were “very applicable” and “fairly applicable” were “Emotional dependence on pediatrics” with 80 cases (37.7%)/101 cases (47.6%) and “Patient's intellectual disability” with 98 cases (46.2%)/76 cases (35.8%), followed by “rare disease” with 75 cases (35.4%)/96 cases (45.3%) (Figure 1-1). Among family factors, “Emotional dependence on Pediatrics” was the highest at 82 cases (38.0%)/108 cases (50.0%), followed by “Over-involvement of patients” at 50 cases (23.1%)/102 cases (47.2%) and “Lack of information about adult departments” at 40 cases (18.5%)/113 cases (25.5%) (Figure 1-2).

**Figure 1-1:**
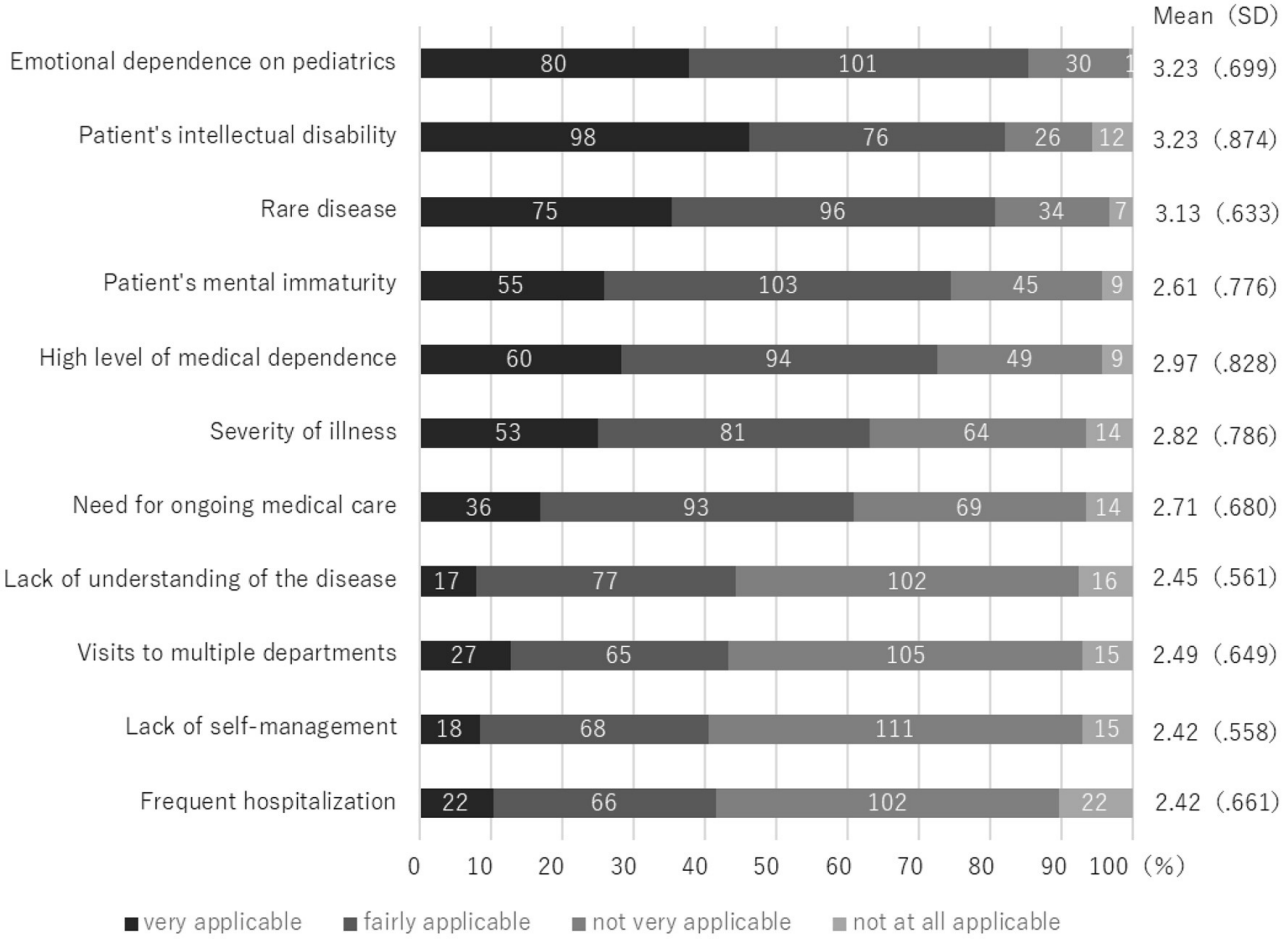
Barrier to Transition from Pediatric to Adult care: Patient Factors (*n* = 212).

**Figure 1-2:**
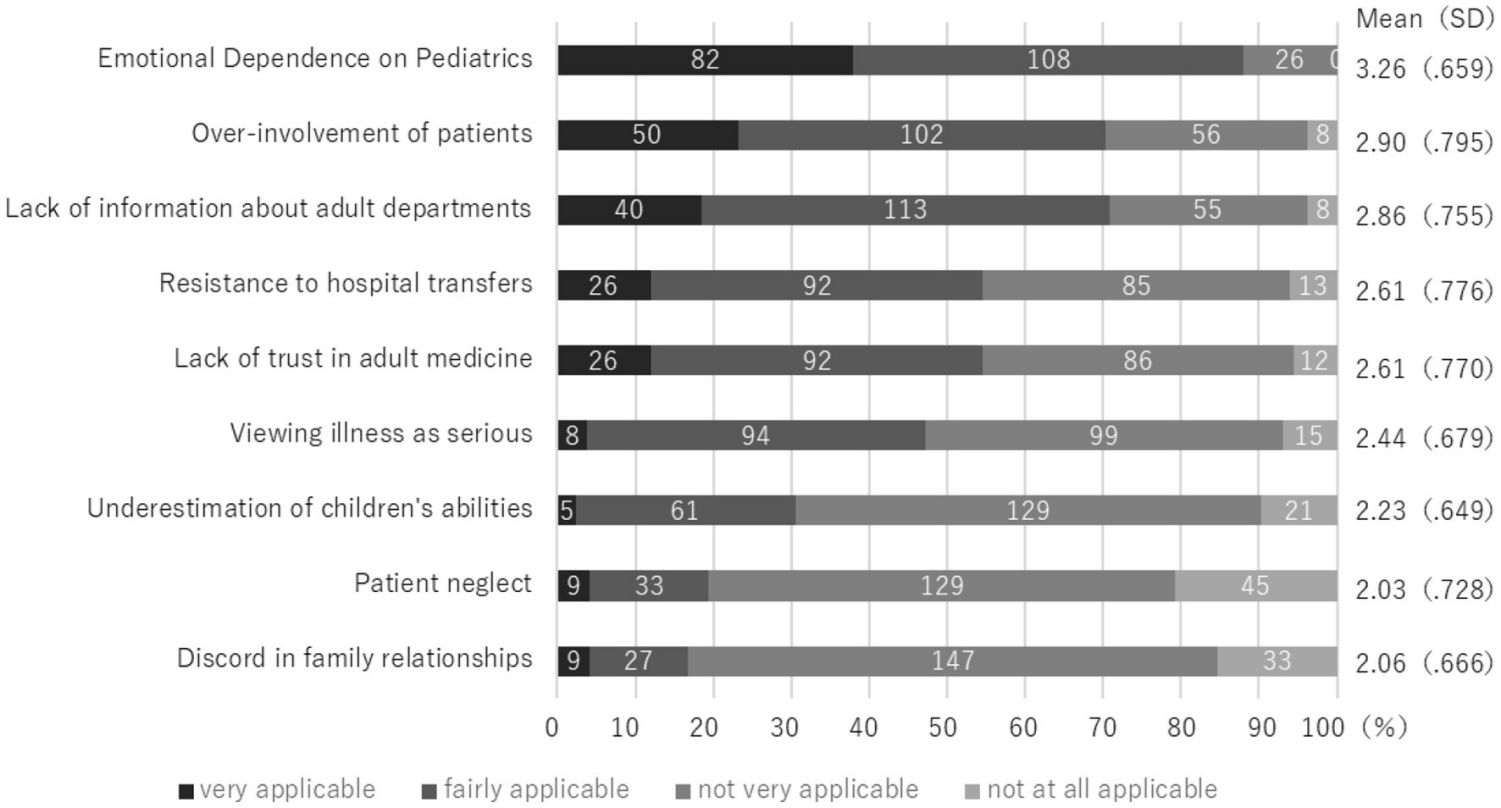
Barriers to Transition from Pediatric to Adult Care: Parents Factors (*n* = 216).

The medical/infrastructure factor with the highest score was “Lack of adult medicine departments” with 86 cases (41.7%)/87 cases (42.2%). The followes were “Lack of understanding of patients and diseases by adult physicians” with 67 cases (32.5%)/100 cases (48.5%), “Lack of personnel to coordinate” with 53 cases (25.7%)/101 cases (49.0%), “Lack of collaboration between pediatricians and adult physicians” 47 cases (22.8%)/107 cases (51.9%), and “Lack of a collaborative system” with 51 cases (24.8%)/95 cases (46.1%) (Figure 1-3).

**Figure 1-3:**
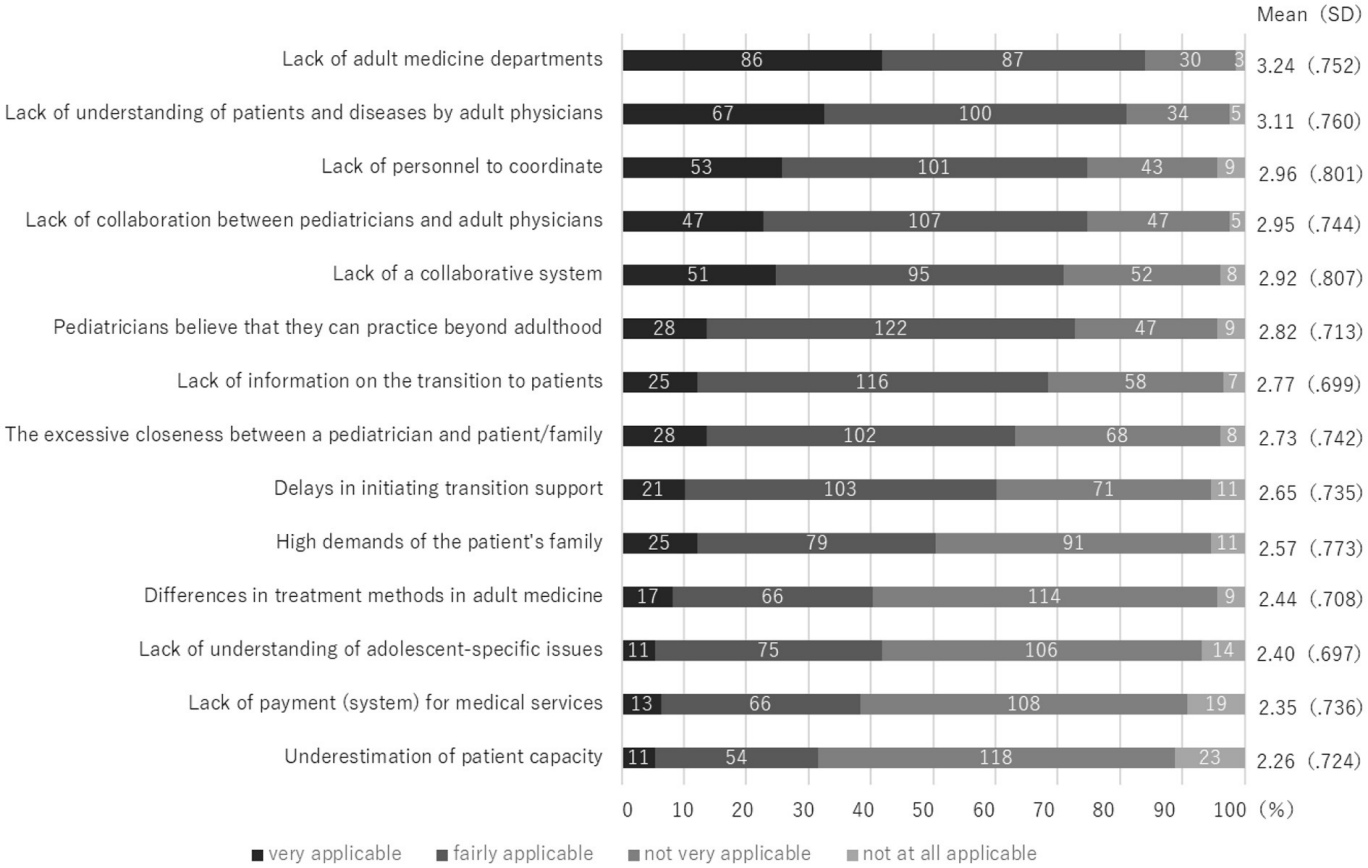
Barriers to Transition from Pediatric to Adult care: Medical/Infrastructure factors (*n* = 206).

### Transitional support for adolescent patients that should be enhanced in the future

The most common item was related to academic institutions, with 166 (73.8%) selecting “sharing knowledge and support methods with adult medicine departments.” This was followed by “establishment of transitional care centers by policies and local governments” with 121 respondents (54.3%) and “securing an adult department to treat adolescent patients” with 114 respondents (51.1%), representing more than half of the sample (Table 4).

**Table 4:** Requests for transition support (Multiple Responses) *n* = 223.

**Unit**	**Content**	***n***	**%**
Academic	Sharing knowledge and support methods with adult medicine departments	166	73.8
	Development of HCT program and guidelines	91	40.8
	Public Awareness Activities	90	40.4
	Advocacy of core concepts	32	14.3
Policy & municipalities	Establishment of transitional care support centers	121	54.3
	Medical expense subsidies for patients	96	43.0
	Revision of medical fees	90	40.4
	Employment support for patients	87	39.0
Institution	Securing an adult department to treat adolescent patients	114	51.1
	Securing human resources	79	35.4
	Creation of departments (divisions)	69	30.9
	Educate and inform patients and families	52	23.3
	Educate and inform staff	49	22.0
	Secure budget	39	17.5
	Sharing the goal philosophy	17	7.6
	Survey of current patient status	13	5.8
Individuals	Communicate and share information with the adult department	95	42.6
	Acquisition of knowledge and expertise in support	80	35.9
	Coordination for transfer to the adult department	61	27.4
	Checking readiness of patient for transition	53	23.8
	Developing a care plan	34	15.2
	Follow up with patients after transfer	22	9.9
	Prepare transition summary	19	8.5
	Evaluation of transition support	11	4.9

## Discussion

### Characteristics of the respondents

The survey's respondents were pediatricians and nurses in management positions at major pediatric institutions in Japan, and their views may reflect the principles and conditions of practice applicable to transition of care in adolescents and young adult health care in Japan.

In terms of specialties, “neuromuscular diseases” and “syndromes involving chromosomal or genetic changes” accounted for about half of the cases. Although a systematic approach to transitional support for neurological diseases is currently being developed in the U.S. (33), the current situation in Japan is not yet fully understood and programs must be developed to enhance support in the future.

Compared with previous surveys on HCT (34–37), the response rate for this survey (45%) was standard for national surveys that were not limited to board-certified physicians or medical departments. About half of the facilities that did not respond might understand HCT but did not provide support or did not have a sufficient understanding of the HCT available.

### Status of HCT programs in Japan

The results of this study showed that although more than 80% of adolescent patients were being transferred to adult departments, few departments and people were dedicated to transition support, and educational and informational tools were not being used. Regarding the HCT contents based on the Six Core Elements, the most common is transferring the patient, followed by the making of a medical summary, with 30–40% of the medical summaries containing information on the disease, and <5% related to patient's understanding of their disease or self-management. Regarding the HCT process based on the Six Core Elements, transfers accounted for about half of the cases. The purpose of the HCT program is not limited to transferring, but to provide seamless, high-quality, and developmentally appropriate medical services during the developmental process from adolescence to adulthood to maximize a person's role functioning and potential (29, 30).

Therefore, developing a transition plan with a medical summary in a transition program should not end with simply sending medical information to the adult department, but should include a transition summary (31). In the Transition to Adult Care program for sickle cell disease, the medical summary includes not only medical information, but also social, academic, and emotional content sent by the nurse case manager (38). The medical summary is also used as a tool to engage the patient or family in taking ownership of medical care (39). One method is an initiative that allows patients, pediatricians, and adult health care providers to share an electronic medical summary website (40). These may help bridge the gap between pediatric and adult care and are important in achieving a seamless transition. Development and research of tools that can be shared longitudinally and with patient families is needed in Japan.

About 15% of the facilities had HCT programs that combined four or more elements. The Six Core Elements are not a model of care, but a structured process. They can be customized for each hospital's use and can be applied to different types of transitional care models and settings (31). The structured HCT processes have shown positive results in reducing pre-transition patient anxiety and enhancing patients' experiences and satisfaction with their care, and interventions that had positive outcomes were described as having a combination of HCT activities (17, 41–43). These findings indicate a need for more widespread implementation of structured HCT programs in Japan.

### Patient and family factors hindering the transition to adult care

In this study, the top factors that prevented patients from transitioning to adult care were intellectual disability and rare diseases among patient factors and emotional dependence on pediatrics among patient and family factors. Similar findings have been reported in Japan and other countries (25, 38, 44). Patient and family factors in transition barriers are said to include anxiety about transition, inadequate planning, and systemic problems (31), indicating that there are compounding factors. These considerations indicate that a combination of structured processes is needed to provide support.

As for rare diseases and intellectual disabilities, in Japan, the percentage of patients over the age of 20 with congenital metabolic disorders exceeds 35% (45), and children with rare diseases are reaching adulthood. The importance of supporting children with chronic illnesses who require these special considerations and the need for research is described (38), and the needs of patients and families (46, 47), barriers to transition (48), clinical reports on support (49–51), informational sites (52, 53), pediatric and adult department practices, and consensus on pediatric and adult medical care and support have been reported (54). Thus, it is necessary to study the current situation and support patients with special medical needs transitioning to adult care in Japan in the future.

Ochiai, in a survey of patients 15 years of age and older visiting a pediatric cardiology outpatient clinic of a children's hospital, described a lack of information about transfers and the need for continued attendance at a pediatric hospital (32). Based on such surveys, we developed the questionnaire regarding patient/family barriers, but they may not cover all of them.

### Expectations for transitional medical support centers

The results of this survey showed that less than half of the respondents were collaborating with adult health care, and challenges to collaborating with adult health care were identified as a factor hindering transition. Also, the top expectations for transition support were related to collaborating with adult health care. The challenges in collaborating with adult and pediatric departments in Japan included the following: lack of communication/systems, lack of understanding and knowledge of pediatric care and patient characteristics by adult health care departments, lack of manpower/institution to coordinate, and difficulties in securing an adult department to treat adolescent patients. Research reports on barriers to transition in the health care system include communication and consultation gaps, knowledge and training limitations, lack of personnel and resources, and financial constraints (23, 31, 55, 56), similar to those found in this study.

One way to resolve these issues, at the policy and municipal levels, is to “establish transitional medical support centers.” This is a facility that provides comprehensive support for transition, including not only medical care but also welfare. In 2017, a model project was launched by the Ministry of Health, Labor and Welfare requiring each prefecture to secure at least one transitional medical support center (57), but as of 2021, there were only seven such centers nationwide. Their main roles include the following: (1) identification and publication of information on medical departments and medical institutions that can treat patients with chronic pediatric diseases in adulthood; (2) liaison, coordination, and communication support between pediatric medical institutions and adult medical institutions; and (3) promotion of support for patients' independence and autonomy (57). It is expected that the establishment of this support organization will be expanded in the future.

In Japan, various physicians' professional organizations developed guidelines for congenital heart disease (58), renal disease (59), type 1 diabetes (60), rheumatic diseases (61), and various other diseases. In the field of adult congenital heart disease in Japan, close affiliation and interaction with the International Society for Adult Congenital Heart Disease and European meeting in Adult Congenital Heart Disease exists, and a system of medical care and certified physicians has been introduced (62). In other countries, advanced practice nurses in charge can provide care that meets patients' needs (63), and they also improve patient care and family satisfaction (64). Thus, it is necessary to train specialists without being limited to a specific department to ensure that patients receive seamless transition support.

In Japan, few facilities have transition coordinators (36) and pediatricians are responsible for most of them. Although there are some projects and organizations that provide transition coordinator training programs (65) and ongoing training for pediatric nurses (66), there are no systematic educational programs. In other countries, there are already educational systems for pediatric and adult health care providers (67, 68). Therefore, in Japan, it is desirable to harmonize and improve the quality-of-care delivery through education and knowledge sharing in the future.

### Limitations of this study and future issues

The results of this survey were mainly derived from physicians nationwide, and we believe that the status of transitional support in pediatric care is clear; however, because facilities that provide transitional support were more likely to respond, those that did not respond to the survey may not be providing adequate support. Therefore, the full scope of support, including that of adult departments and patients/families, might not have been captured. It would therefore be necessary to continue the survey by expanding its scope and considering specific transitional care and support.

The results of this survey were mainly derived from physicians nationwide, and we believe that the status of transitional support in pediatric care is clear. We found that about half of the facilities do not provide transition support due to barriers or insufficient understanding of specific support. As a future challenge, we believe it is necessary to make new contacts with facilities that did not respond to the survey and learn about their difficulties in promoting a systematic HCT program.

## Conclusions

We sent self-administered questionnaires to pediatricians and nurses in 494 facilities throughout Japan and received responses from 225 facilities, of which approximately 80% had implemented “transitioning patients.” However, the structured implementation of transition programs was not standardized. Barriers to transition related to the medical institutions included a lack of coordinators and difficulties collaborating with adult departments due to a lack of adult departments that could handle pediatric conditions. Patient/family-related barriers to transition included delayed independence due to disability and psychosocial factors, as well as lack of information about the HCT. To resolve these issues, it is suggested that transition support be developed according to the characteristics of rare diseases and disabilities, transition medical care support centers be popularized, coordinators be appointed as a support system, and a system of collaboration between pediatric and adult departments be established.

## Data availability statement

The raw data supporting the conclusions of this article will be made available by the authors, without undue reservation.

## Ethics statement

The studies involving human participants were reviewed and approved by Saitama Prefectural University Research Ethics Committee. The patients/participants provided their written informed consent to participate in this study.

## Author contributions

IS, MM, TM, and MH contributed to conception and design of the study. IS organized the database, performed the statistical analysis, and wrote the first draft of the manuscript. MM wrote sections of the manuscript. All authors contributed to manuscript revision, read, and approved the submitted version.

## Funding

This research is part of the Japanese Ministry of Education, Culture, Sports, Science and Technology (MEXT) Grant-in-Aid for Scientific Research, Basic C (Grant #19K11036, PI Ikuho Sakurai) for the 2019–2022 fiscal year.

## Conflict of interest

The authors declare that the research was conducted in the absence of any commercial or financial relationships that could be construed as a potential conflict of interest.

## Publisher's note

All claims expressed in this article are solely those of the authors and do not necessarily represent those of their affiliated organizations, or those of the publisher, the editors and the reviewers. Any product that may be evaluated in this article, or claim that may be made by its manufacturer, is not guaranteed or endorsed by the publisher.
